# Changes in spinal alignment one month post abdominal surgery: A prospective cohort study

**DOI:** 10.1097/MD.0000000000033674

**Published:** 2023-04-28

**Authors:** Akihiro Ito, Shinno Iijima

**Affiliations:** a Department of Physical Therapy, School of Health Science, International University of Health and Welfare, Otawara, Japan; b Department of Physical Therapy and Rehabilitation, International University of Health and Welfare Hospital, Nasushiobara, Japan.

**Keywords:** digestive system surgical procedures, pain, perioperative period, posture, spine

## Abstract

Following abdominal surgery, many patients assume a bending or stooping posture to protect the surgical wound. Such postural changes are known to induce side effects, but the improvement and persistence of these effects are unknown. Therefore, the purpose of this study was to clarify the nature of postural changes in patients undergoing abdominal surgery. In this prospective cohort study, we enrolled 25 patients who underwent abdominal surgery from February 2019 to January 2020. Measurements were obtained during the preoperative, pre-discharge, and first outpatient stages. The sacral tilt, lumbar lordotic, thoracic kyphosis, and overall tilt angles were measured in the static standing position in a private room. Wound pain was measured using a Visual Analogue Scale. Repeated measures analysis of variance was applied to compare spine measurements for each measurement period, followed by the Bonferroni method for each level. Pearson’s product-moment correlation coefficient was performed to examine the relationship between wound pain and spinal column angle. The lumbar kyphosis angle was lower before discharge (−7.2 ± 7.4°) compared to preoperatively (−11.1 ± 7.5°) (95% CI 0.76, 7.08; *P* < .01, η^2^ = 0.21). Regarding the overall tilt angle, the anterior tilt angle increased before discharge (3.4 ± 3.9°) compared to preoperatively (1.1 ± 4.1°) (95% CI 0.86, 3.78; *P* < .01, η^2^ = 0.33). No statistically significant correlation with pain was observed. Compared to the preoperative period, the patients had an anterior tilt, mainly due to lumbar spine changes, prior to discharge from the hospital. Changes in spinal alignment were not associated with wound pain.

## 1. Introduction

The International Agency for Research on Cancer has reported that colorectal cancer is the third most common cancer worldwide, while stomach cancer is the fifth most common.^[1]^ The number of abdominal surgeries is increasing, especially in East Asian countries such as Japan due to the large number of cancer patients.^[2]^ The most common complication following abdominal procedures is pain centered around the surgical wound, and many reports regarding its intensity, changes, associations with cancer site,^[3,4]^ surgical technique,^[5]^ and long-term duration have been published.^[6]^ Various treatment strategies for postoperative pain have been investigated including reducing the release of inflammatory cytokines through preoperative nutritional therapy,^[7]^ intraoperative anesthetic administration,^[8]^ and application of postoperative analgesics,^[9,10]^ among others. One simple, noninvasive, and low-cost method that patients can perform independently to protect their postoperative wounds is to adopt an anterior tilt posture. The forward-leaning posture is most commonly observed in clinical situations and is believed to reduce stretching and pressure on the skin surrounding the wound, thereby preventing the occurrence and increases of pain. However, it has been reported that a flexed posture leads to decreased respiratory function,^[11,12]^ back muscle strength,^[13]^ and grip strength,^[14]^ resulting in postoperative complications,^[15]^ back pain,^[16]^ decreased walking ability,^[17]^ and falls from impaired balance.^[18]^ As such, it is important to promptly improve the anterior tilt posture and address its causes. However, the only studies that have examined postoperative postural changes have been for neck^[19,20]^ and chest surgery,^[21,22]^ and no studies have focused on postoperative postural changes following abdominal surgery. Furthermore, no studies have examined how long postoperative postural changes last or whether they improve spontaneously. Clarification of these factors would be an important indicator for improving physical function and providing patient guidance. Therefore, in the present study, we aimed to track postural changes in patients who had undergone abdominal surgery for > 1 month and to clarify the characteristics of these changes and their transitions. We hypothesized that patients would lean forward postoperatively, predominantly at the lumbar region to protect the abdomen, but that this would improve with pain reduction. We believe that the clarification provided by this study will be useful in optimizing postoperative wound protection and subsequent exercise therapy.

## 2. Methods

### 2.1. Participants and procedure

This study had a prospective cohort design and was conducted with patients who underwent abdominal surgery on a standby basis from February 2019 to January 2020 at the Department of Gastroenterology, International University of Health and Welfare Hospital, Japan. The inclusion criteria were willingness to participate in the study, eligibility to undergo open or laparoscopic surgery for gastrointestinal cancer, and 20 years of age or older. The exclusion criteria included a lack of independence in activities of daily living, a history of spinal or neurological disease, and inability to maintain a standing position.

A power analysis was conducted to assess how many patients should be included in the study. An obstacle in determining the effect size was the lack of literature comparing the first outpatient visits before and after surgery as well as after discharge. Therefore, we used partial η^2^ effect sizes in the “medium” and “large” ranges of 0.06 and 0.14. Using a *P* value of .05, a beta of 0.8, and a correlation of 0.5, we calculated the required sample size to be 16 to 27. To account for the possibility that half of the patients would meet the exclusion criteria or be lost to follow-up, the expected number of participants was set at 40.

Perioperative rehabilitation includes the preoperative assessment of physical function, explanation of the postoperative rehabilitation program, respiratory training to prevent postoperative complications, and exercise therapy to maintain physical function. Following surgery, patients consulted with the attending physician regarding getting out of bed, which began on the first postoperative day. Activities of daily living was increased in a stepwise fashion, and patient movements were guided, with wound pain and use of the muscles surrounding the drain insertion site taken into particular consideration. In addition, routine pulmonary toilet techniques were provided, including breathing treatments, postural drainage, and percussion, as indicated.

This study was approved by the Ethics Review Committee of the International University of Health and Welfare (approval number: 18-Io-81). This study was conducted in accordance with the guidelines outlined in the Declaration of Helsinki. The participants were informed of the content and purpose of the study in advance, and consent was obtained both orally and in writing.

Patient background information including conditions leading to surgery, cancer stage, and complications was investigated. Perioperative data, including procedure type, duration of surgery, duration of epidural anesthesia, and use of analgesics at the time of pre-discharge measurement, were also obtained from the medical records.

### 2.2. Assessment

Measurements were taken by a physical therapist; the results were analyzed by a separate individual. Measurements were taken before surgery, before discharge (1 week after surgery), and at the first outpatient visit after discharge (4 weeks after surgery). Spinal alignment measurements were performed using Spinal Mouse (Idiag AG Corp., Zurich, Switzerland) to examine postural changes. Spinal Mouse is a device that can measure spinal kyphosis angles non-invasively using a 3-axis accelerometer. Mannion et al^[23]^ and Kellis et al^[24]^ previously investigated the reproducibility and reliability of the Spinal Mouse and found that both the intra- and inter-rater reliability were greater than 0.8. The validity of the measured values has also been compared with the values measured from X-ray images, and a high correlation was found.^[23]^

The measurement limb position was arranged from a stationary standing position: the participant was placed with both feet parallel to each other, with a foot width of 10 cm between the medial malleolus and both upper limbs, in a spontaneous drooping position. To establish the head position, the participant was asked to gaze forward at eye level. In each limb position, the Spinal Mouse was placed on the paraspinal line from the seventh cervical vertebra to the third sacral vertebra, and measurements were taken in the craniocaudal direction. The results were calculated as follows: sacral tilt angle (anterior tilt +, posterior tilt −), lumbar lordotic angle (lordosis −, kyphosis +), thoracic kyphosis angle (lordosis −, kyphosis +), and the overall tilt angle (anterior tilt +, posterior tilt). Additionally, wound pain was assessed using a visual analogue scale.

### 2.3. Statistical analysis

Normality was assessed using the Shapiro–Wilk test. Repeated measures ANOVA was subsequently performed to compare each spinal measurement at the preoperative, pre-discharge, and first post-discharge outpatient visits, and multiple comparisons were made between each level using the Bonferroni method. To examine the relationship with wound pain, Pearson’s product rate correlation coefficient analysis was performed between the pre-discharge and initial post-discharge outpatient angles and wound pain. Effect sizes were calculated by post hoc testing after each trial. Statistical analyses were performed using SPSS version 25 (IBM Corp., Armonk, NY) for all comparative analyses. Statistical significance was set at *P* < .05.

## 3. Results

**Figure 1.**
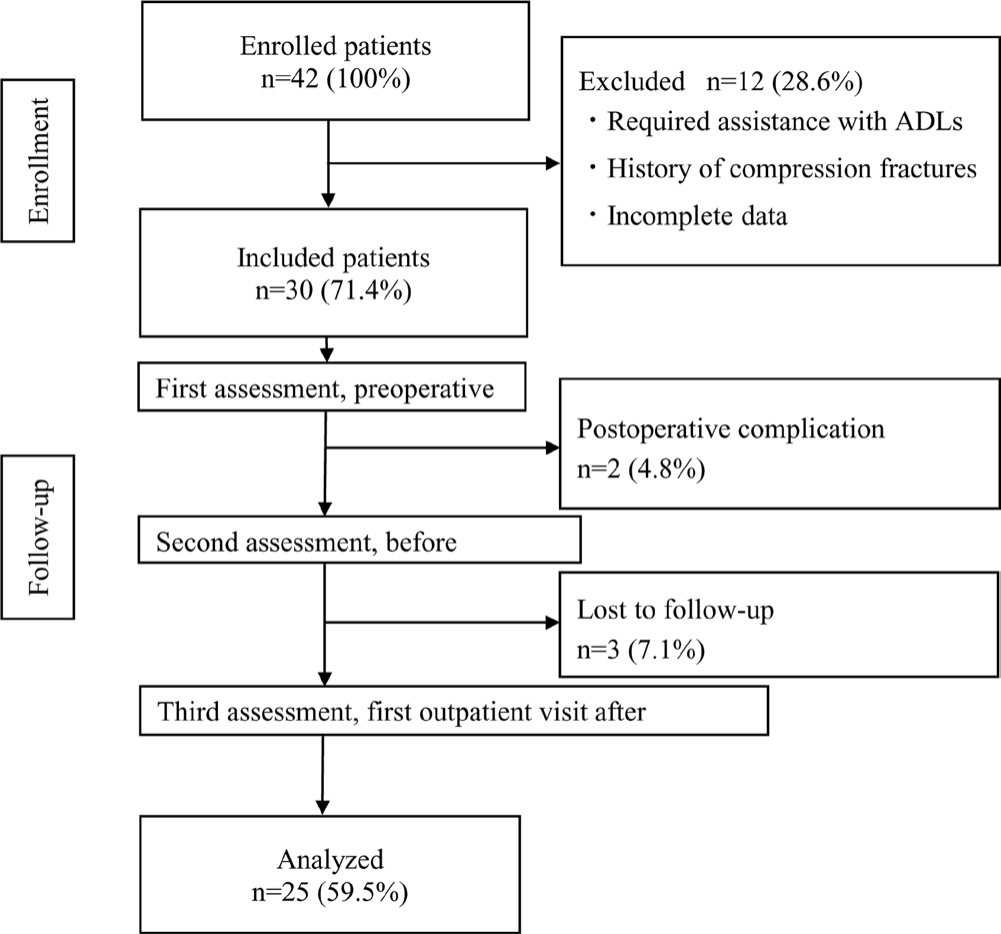
Flow-chart of patient recruitment for this study. ADL = activities of daily living.

In total, 42 participants were initially included in the study. Of these, 30 patients who met the study criteria were included. Of the 30 eligible patients, 2 were excluded from the study due to postoperative complications and 3 were unable to complete the follow-up evaluation. Finally, 25 patients were included in the analysis. Figure 1 illustrates the participant selection process, and Table 1 shows the participant characteristics. The timing of each postoperative measurement was 7.3 ± 2.2 days at discharge and 26.6 ± 5.7 days at the first outpatient visit after discharge.

**Table 1:** Participant characteristics (n = 25).

Range of ages	60–69 (n = 12)	70–79 (n = 9)	80≥ (n = 4)
Age (yr) (mean ± SD)	65.0 ± 2.8	73.4 ± 2.7	81.5 ± 1.3
Sex, n (%)			
M	9 (75.0)	6 (66.7)	3 (75.0)
F	3 (25.0)	3 (33.3)	1 (25.0)
Height (cm) (mean ± SD)	164.6 ± 8.0	159.0 ± 10.3	161.7 ± 3.5
Weight (kg) (mean ± SD)	65.9 ± 11.2	61.3 ± 7.5	58.2 ± 8.6
Body mass index (kg/m^2^) (mean ± SD)	24.3 ± 3.6	24.3 ± 2.8	22.2 ± 3.2
Preoperative diseases			
Disease state, n (%)			
Stomach cancer	3 (25.0)	3 (33.3)	2 (50.0)
Sigmoid colon cancer	3 (25.0)	1 (11.1)	0 (0.0)
Cecal cancer	1 (8.0)	2 (22.2)	0 (0.0)
Ascending colon cancer	1 (8.0)	0 (0.0)	1 (25.0)
Transverse colon cancer	1 (8.0)	1 (11.1)	0 (0.0)
Rectal cancer	1 (8.0)	1 (11.1)	0 (0.0)
Esophageal cancer	1 (8.0)	0 (0.0)	0 (0.0)
Descending colon cancer	0 (0.0)	0 (0.0)	1 (25.0)
Liver tumor	0 (0.0)	1 (11.1)	0 (0.0)
Gallbladder cancer	1 (8.0)	0 (0.0)	0 (0.0)
Stage, n (%)			
Ⅰ	5 (41.7)	4 (44.4)	2 (50.0)
Ⅱ	3 (25.0)	3 (33.3)	2 (50.0)
Ⅲ	4 (33.3)	2 (22.2)	0 (0.0)
High blood pressure, n (%)	4 (33.3)	3 (33.3)	2 (50.0)
Hyperlipidemia, n (%)	2 (16.7)	0 (0.0)	1 (25.0)
Diabetes, n (%)	4 (33.3)	3 (33.3)	0 (0.0)
Postoperative characteristics			
Type of surgery, n (%)			
Laparoscopic surgery	10 (83.3)	8 (88.9)	3 (75.0)
Open surgery	2 (16.7)	1 (11.1)	1 (25.0)
Formation of stoma	1 (8.0)	0 (0.0)	0 (0.0)
Surgery time (min) (mean ± SD)	262.5 ± 120.6	274.0 ± 95.3	293.25 ± 123.0
Postoperative hospital stay (d) (mean ± SD)	11.5 ± 7.5	14.4 ± 9.2	13.8 ± 6.7
Epidural analgesic withdrawal date (d) (mean ± SD)	4.6 ± 1.4	3.9 ± 1.5	4.8 ± 0.5
Medication status at time of pre-discharge measurement, n (%)			
Celecoxib	0 (0.0)	3 (33.3)	2 (50.0)
Oxoprofen Sodium Hydrate	4 (33.3)	0 (0.0)	0 (0.0)
Acetaminophen	1 (8.0)	2 (22.2)	0 (0.0)
Fentanyl Citrate	2 (16.7)	0 (0.0)	0 (0.0)
Pentazocine	0 (0.0)	1 (11.1)	0 (0.0)
Diclofenac Sodium	1 (8.0)	0 (0.0)	0 (0.0)

Overlaps exist for disease status, type of surgery, and analgesic medications.

The changes in spinal angles are shown in Table 2. The lumbar lordotic angle showed a decrease in lordotic angle prior to discharge (−7.2 ± 7.4°) compared to the preoperative angle (−11.1 ± 7.5°) (95% CI 0.76, 7.08; *P* < .01, η^2^ = 0.21). The anterior tilt angle increased before discharge (3.4 ± 3.9°) compared to the preoperative measurements (1.1 ± 4.1°) (95% CI 0.86, 3.78; *P* < .01, η^2^ = 0.33).

**Table 2:** Results of repeat measures ANOVA of spinal alignment at each measurement.

	Preoperative	Before discharge	First outpatient visit after discharge
Sacral tilt angle (°) (Mean ± SD)	−0.2 ± 6.3	−0.6 ± 6.8	−0.6 ± 6.1
Thoracic kyphosis angle (°) (Mean ± SD)	45.1 ± 8.3	42.1 ± 9.9	43.8 ± 10.0
Lumber lordotic angle (°) (Mean ± SD)	−11.1 ± 7.5*	−7.2 ± 7.4*	−9.8 ± 7.7
Overall tilt angle (°) (Mean ± SD)	1.1 ± 4.1*	3.4 ± 3.9*	2.0 ± 3.6

* Bonferroni test was used.

**Figure 2.**
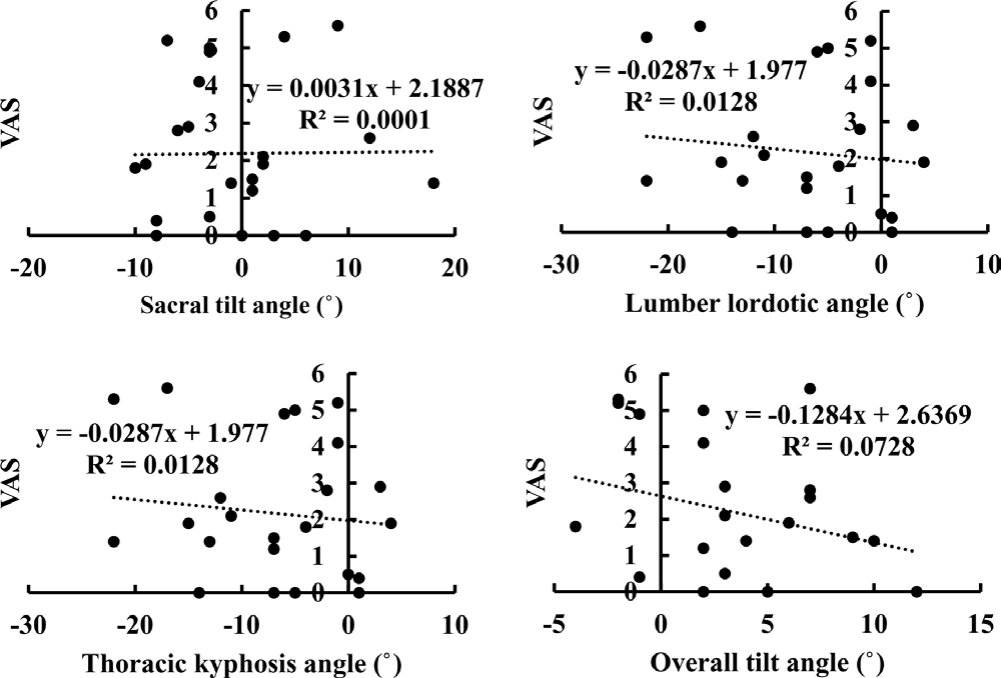
Scatterplot of spinal alignment and pain before discharge. VAS = visual analogue scale.

**Figure 3.**
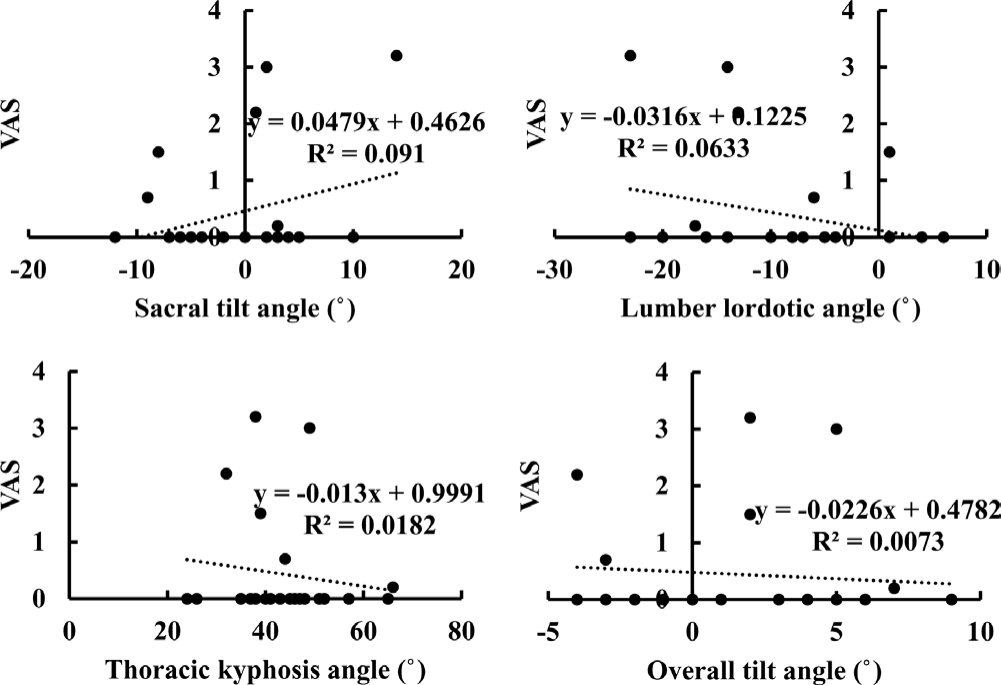
Scatterplot of spinal alignment and pain at initial outpatient visit. VAS = visual analogue scale.

At the first outpatient visit, the visual analogue scale score for postoperative wound pain was 2.2 ± 1.9 pre-discharge and 0.5 ± 1.0. Table 3; Figures 2 and 3 show the correlation between the site of change and wound pain. No significant correlations were found for any item.

**Table 3:** Results of correlation analysis between the spinal angle and pain at discharge and first outpatient visit.

	Visual analogue scale	
	Before discharge	First outpatient visit
Before discharge		
Sacral tilt angle (°)	0.055	
Lumber lordotic angle (°)	−0.131	
Thoracic kyphosis angle (°)	−0.114	
Overall tilt angle (°)	−0.232	
First outpatient visit		
Sacral tilt angle (°)		0.302
Lumber lordotic angle (°)		−0.252
Thoracic kyphosis angle (°)		−0.135
Overall tilt angle (°)		−0.085

Correlation coefficients between each indicator are shown.

## 4. Discussion

This is the first study to investigate postoperative postural changes in patients following abdominal surgery. Overall, the results showed that the predominant spinal alignment following abdominal surgery was an anteverted posture, which was primarily caused by changes in the lumbar curve. The forward-leaning posture improved over time, but was not always completely improved even after 4 weeks. Furthermore, we found no correlation between the changes in spinal alignment and wound pain.

It has previously been suggested that surgical invasion may have a strong impact on the nearest spinal site. Therefore, we believe that changes in the lumbar spine were observed in study participants who underwent abdominal surgery. Even in non-abdominal surgery, it has been reported that changes in the kyphosis angle of the upper cervical spine can be observed following middle and lower cervical spine surgery, while thoracic changes can be observed after cervical surgery.^[19,20]^ Thoracic cage and thoracic spine surgery have been shown to induce changes in shoulder joint range of motion and thoracic spine kyphosis angle.^[21,22]^ Furthermore, hip surgery has also been reported to affect the lumbar spine and pelvic mobility.^[25,26]^

Although changes in spinal alignment, particularly those in the lumbar spine, were observed, no association was found with wound pain. Postoperative wound pain is experienced by many patients and has been reported to be associated with various postoperative complications.^[27]^ However, we found no association between wound pain and spinal alignment in the present study. In addition to pain, surgical wounds may affect spinal alignment due to inflammation and scarring.^[28]^ Therefore, other perioperative factors besides pain should be investigated to determine the factors that contribute to postoperative changes and subsequent improvements in spinal alignment.

The inclusion and exclusion criteria of the present study were designed so that the effects of abdominal surgery could be isolated for assessment. However, this study has several limitations that must be acknowledged. First, it was performed in a single center and had a small number of patients. Therefore, it was not possible to collect a sufficient number of patients for a detailed comparison of each technique, and a larger number of patients is needed for generalization. Second, factors that affect spinal alignment other than wound pain were not examined. In addition to wound pain, some patients also complained of skin scarring and anxiety during movement, which could be considered factors for change and should be examined in more detail. Lastly, the living situation after discharge from the hospital and the amount of activity were unknown. No restrictions were placed on the amount of movement or activity in the patient’s post-discharge life, and individualized investigations were not conducted. Thus, it is possible that the type of activities performed and the amount of activity may have influenced improvement.

In the future, it will be necessary to clarify the relationship between postural changes and physical functions, as well as the methods and effects of physical therapy, based on the abovementioned points. The results of such an analysis would be more useful in clinical practice.

## Acknowledgments

We would like to thank the faculty members of the Digestive Surgery and Rehabilitation Office at the International University of Health and Welfare for their advice and guidance. We would also like to thank the participants for their cooperation.

## Author contributions

**Conceptualization:** Akihiro Ito, Shinno Iijima.

**Data curation:** Akihiro Ito, Shinno Iijima.

**Formal analysis:** Akihiro Ito, Shinno Iijima.

**Funding acquisition:** Akihiro Ito.

**Investigation:** Shinno Iijima.

**Methodology:** Akihiro Ito, Shinno Iijima.

**Project administration:** Akihiro Ito.

**Resources:** Akihiro Ito.

**Software:** Akihiro Ito.

**Supervision:** Akihiro Ito.

**Validation:** Akihiro Ito.

**Visualization:** Akihiro Ito.

**Writing – original draft:** Akihiro Ito.

**Writing – review & editing:** Shinno Iijima.
